# Combined Electromagnetic Fields Mitigate Unloading-Induced Bone Loss by Enhancing Osteogenic Responses via Multiphysics-Induced Mechanotransduction

**DOI:** 10.3390/cells15131138

**Published:** 2026-06-23

**Authors:** Chao Cai, Shenghang Wang, Junyu Liu, Mengxuan Zheng, Weihao Ren, Fengyi Xue, Xin Zhang, Bo Zong, Jiancheng Yang, Weikang Sun, Zhihua Li, Tinghua He, Xiaotong Zhang, Peng Shang

**Affiliations:** 1 Research & Development Institute in Shenzhen, Northwestern Polytechnical University, Shenzhen 518057, China; caichao@mail.nwpu.edu.cn (C.C.); wangshenghang@uhrs.edu.cn (S.W.); liujunyu@mail.nwpu.edu.cn (J.L.); renweihaomk@163.com (W.R.); xuefy@mail.nwpu.edu.cn (F.X.); xinzhang0905@163.com (X.Z.); zongbo@mail.nwpu.edu.cn (B.Z.); sunwk@mail.nwpu.edu.cn (W.S.); lzhxby@mail.nwpu.edu.cn (Z.L.); hth.nwpu.edu.cn@mail.nwpu.edu.cn (T.H.); 2 School of Life Science and Technology, Northwestern Polytechnical University, Xi’an 710129, China; 3 Shandong Key Laboratory of Neurorehabilitation, Shandong Engineering Research Center of Precision Intervention for Aging, School of Life Sciences and Health, University of Health and Rehabilitation Sciences, Qingdao 266113, China; 4 College of Information Science and Engineering, Northeastern University, Shenyang 110819, China; zhengmengxuan@ise.neu.edu.cn; 5 Department of Osteoporosis, Honghui Hospital, Xi’an Jiaotong University, Xi’an 710054, China; yangjiancheng@nwpu.edu.cn; 6 College of Electrical Engineering, Zhejiang University, Hangzhou 310027, China

**Keywords:** bone formation, combined electromagnetic field (CEMF), mechanical stimulation, hindlimb unloading (HLU), PI3K-AKT, physical therapy

## Abstract

Unloading-induced bone loss is a major medical challenge during long-duration human spaceflight, largely driven by suppressed osteoblast-mediated bone formation, and practical countermeasures are needed. Electromagnetic stimulation has shown benefits for bone repair, and its non-invasiveness supports potential space use; however, its single-modality efficacy remains limited. Here, we investigated a combined electromagnetic field (CEMF) integrating a static magnetic field (SMF, 0.4–0.6 T) and a pulsed electromagnetic field (PEMF, 0.38 ± 0.19 mT) to attenuate unloading-related bone loss and examine field-induced mechanical stimulation. Finite-element simulations mapped magnetic flux density, field gradient, induced current density, and Lorentz force density in bone tissue. CEMF was evaluated in vivo in hindlimb unloading (HLU) mice and in vitro in MC3T3-E1 osteoblasts. CEMF improved bone mineral density, trabecular and cortical microarchitecture, and mechanical properties in HLU mice, with increased osteoblast number and mineral apposition rate. In vitro, CEMF promoted osteogenic differentiation and upregulated COL1A1 and RUNX2. Transcriptome analysis suggested activation of ECM–integrin mechanical signaling and the PI3K–AKT pathway. These findings indicate that CEMF-induced multiphysics stimulation enhances osteogenic responses and may serve as a complementary, non-invasive countermeasure for spaceflight-associated bone loss.

## 1. Introduction

Long-duration spaceflight exposes astronauts to sustained microgravity, resulting in progressive bone loss. Previous space missions have demonstrated that after long-duration spaceflight, significant reductions in bone mass occur in weight-bearing skeletal regions, affecting both cortical and trabecular compartments [1]. This deterioration is associated with decreased bone mineral density, microarchitectural degradation, reduced mechanical strength, and increased fracture risk. Bone loss under microgravity is widely recognized as a result of prolonged mechanical unloading. Under normal gravitational conditions, bone remodeling is a dynamic physiological process maintained by the coordinated coupling of bone formation and bone resorption, in which mechanical stimulation serves a critical regulatory role. Studies have shown that mechanical strain influences osteoblast behavior through mechanotransduction pathways such as the integrin-extracellular matrix axis [2], suppresses osteoclast differentiation signals [3], and inhibits osteoclastic resorption in vitro [4]. Under microgravity conditions, the reduction in mechanical stimulation disrupts this balance. Studies have found that microgravity alters bone cell responses to mechanical stimuli [5], modifies osteoblast gene expression [6,7], and induces pathological processes, including osteocyte apoptosis [8]. These findings indicate that insufficient mechanical signaling is a key contributor to spaceflight-associated bone loss [9].

Current countermeasures primarily rely on resistive exercise devices and artificial gravity concepts, such as self-powered human centrifugation [10]. Although partially effective, these approaches are limited by system complexity, spacecraft constraints, and the difficulty of reproducing physiological multidirectional loading patterns. As future missions extend toward long-duration and deep-space exploration, there is a need for complementary countermeasures that are efficient, compact, and compatible with spacecraft environments [11]. Electromagnetic stimulation is a non-invasive physical intervention for bone-related disorders. The piezoelectric properties of bone [12,13] demonstrated intrinsic electromechanical coupling within bone tissue. Unlike conventional mechanical loading, electromagnetic fields can interact with biological tissues at the cellular level. Static magnetic fields (SMFs) have been reported to attenuate hindlimb unloading (HLU) and ovariectomized (OVX)-induced bone loss [14,15], and enhance osteogenic differentiation of bone marrow mesenchymal stem cells and osteoblasts [16]. Pulsed electromagnetic fields (PEMFs) have been widely applied in the treatment of fractures and osteoporosis by modulating bone metabolism through inductively coupled electrical stimulation [17]. According to the Lorentz force law, electric currents experience Lorentz forces when exposed to an SMF. Therefore, the combination of SMF and PEMF can generate not only magnetic and electrical effects but also secondary mechanical forces through magnetoelectric interactions [18,19]. Nevertheless, the effects of combined electromagnetic fields (CEMF) on unloading-induced bone loss, as well as the mechanisms by which mechanical stimulation generated by coupled SMF and PEMF regulates bone formation, have not been fully explored.

In this study, we developed a CEMF system integrating SMF and PEMF components and evaluated its effects under mechanical unloading conditions. Using the HLU mouse model as a ground-based microgravity analog, we compared the effects of CEMF with single-field stimulation on bone mass, microarchitecture, and mechanical properties. In addition, we examined its effects on osteogenic differentiation and mechanotransduction-related signaling pathways in MC3T3-E1 osteoblasts. This study aims to provide a novel electromagnetic field-based therapeutic strategy that promotes bone formation for the treatment of spaceflight-induced bone loss.

## 2. Materials and Methods

### 2.1. Construction of the CEMF Device

The CEMF device consists primarily of a PEMF generator and an SMF source based on a Halbach array. The PEMF hardware system includes a waveform generator (VC2015H; Shenzhen Victor Hi-Tech Co., Ltd., Shenzhen, China) and an RF signal generator (SSG3021X; SIGLENT Technologies Co., Ltd., Shenzhen, China), which outputs a high-frequency carrier signal modulated into square-wave pulses with a pulse width of 10 ms and a duty cycle of 10%. The PEMF signal is amplified by a power amplifier and delivered to a solenoidal RF coil with a diameter of 15 mm and 20 turns. The system is equipped with a custom-developed graphical user interface (GUI) that enables real-time monitoring of key parameters and ensures a stable current of 1 A during operation. Current and power output data were recorded at 5 s intervals. The SMF is generated by a Halbach array composed of N42m-grade neodymium iron boron (NdFeB) permanent magnets, producing a vertically upward magnetic field. The inner chamber of the magnet has a diameter of 30 mm, with the magnetic flux density within the exposure region being 0.4–0.6 T. During experiments, mouse hindlimbs were fixed and positioned within the inner chamber of the magnet. Similarly, during cell experiments, cells were subjected to the same magnetic field conditions.

### 2.2. Numerical Simulations

Numerical electromagnetic simulations were performed to evaluate the distributions of the PEMF, magnetic force densities ($F_{0}$), and Lorentz force (F) within tissues. The quasi-static solver in SIM4life (Version 7.2; Zurich Med Tech, Zurich, Switzerland) was used to assess PEMF effects in a mouse model, while ANSYS (Version 2022 R1; ANSYS, Inc., Canonsburg, PA, USA) was utilized to analyze SMF distributions. The electrical properties of tissues (e.g., relative permittivity and conductivity) were assigned based on the Gabriel Model [20], while the magnetic permeability of tissues was set equal to that of a vacuum. Magnetic forces, Lorentz forces calculations, and subsequent statistical analyses were conducted using MATLAB (Version 9.11; MathWorks, Natick, MA, USA).

### 2.3. Animal Experiments and Grouping

Male C57BL/6 mice aged 8 weeks (Charles River, Beijing, China) were used in the experiments. The mice were categorized into five groups (6 in each group), and they were randomly assigned as follows: the control group (control), HLU with geomagnetic field (GMF) exposure group (GMF group), HLU with SMF exposure group (SMF group), HLU with PEMF exposure group (PEMF group), and HLU with SMF and PEMF exposure group (CEMF group). Mice were treated with different EMFs for 20 min per day, and tissue samples were collected for analysis after 4 weeks. After the 4-week intervention, the mice were anesthetized for BMD scanning and then euthanized by cervical dislocation.

### 2.4. BMD and BMC Evaluation

Whole-body skeletal scanning was performed using dual-energy X-ray absorptiometry (DEXA; InAlyzer, MEDIKORS Inc., Seongnam-si, Republic of Korea) to determine bone mineral density (BMD) and bone mineral content (BMC) in the bilateral femora and other anatomical regions.

### 2.5. Micro-CT Analysis

Femoral microarchitecture was examined using micro-computed tomography (micro-CT; VivaCT80, SCANCO Medical AG, Bassersdorf, Switzerland). Quantitative analysis was performed with RayTracer to obtain bone volume fraction (BV/TV), trabecular number (Tb.N), trabecular thickness (Tb.Th), trabecular separation (Tb.Sp), cortical area fraction (Ct.Ar/Tt.Ar), and tissue mineral density (TMD).

### 2.6. Biomechanical Examination

Tibial mechanical properties were evaluated by three-point bending. The tibiae were fixed on a mechanical testing machine, and a progressively increasing load was applied to the midshaft at a displacement rate of 1 mm/min until fracture occurred. Load–displacement curves were recorded using Bluehill software (Version 2; Instron, Norwood, MA, USA) and stress–strain relationships and other mechanical parameters were analyzed with MATLAB (Version 9.11; MathWorks, Natick, MA, USA).

### 2.7. Histochemical and Dynamic Histomorphometric Analysis

Femurs were first decalcified, with the decalcifying solution replaced every 2 days for a total of 8 days. The samples were then paraffin-embedded and sectioned at a thickness of 5 μm. Hematoxylin and eosin (H&E) staining was performed to evaluate bone histomorphology. Stained sections were scanned using a digital slide scanner, and the number of osteoblasts per bone surface (N.Ob/BS) was quantified with KFSlideOS software (Version 1.1.2.0; KFBIO, Yuyao, China).

The mineral apposition rate (MAR) was measured using a double calcein labeling method. Calcein (Sigma-Aldrich, St. Louis, MO, USA) was dissolved in sterile saline at a concentration of 7 mg/mL. Mice were labeled with calcein via intraperitoneal injection at a dose of 35 μg/g body weight, 10 and 3 days before sample collection. The two injections were administered at a 7-day interval to allow for double fluorescence labeling. After fixation in 4% paraformaldehyde for 48 h, the femurs were embedded in methyl methacrylate and sectioned into 50 μm-thick sections. Fluorescent calcein labels were observed and recorded using fluorescence microscopy. To minimize measurement bias, fluorescence signals were collected and analyzed from the same anatomical region of the femoral cortex across different groups. Histomorphometric parameters were analyzed using fluorescence microscopy, and ImageJ software was used for quantification. MAR was calculated based on the distance between the two fluorescent labels divided by the 7-day labeling interval.

### 2.8. Cell Culture

MC3T3-E1 osteoblasts, kindly provided by Professor Hong Zhou of The University of Sydney, were maintained in α-MEM (Gibco, Grand Island, NY, USA) supplemented with 2 mM L-glutamine, 10% fetal bovine serum, and 1% penicillin-streptomycin. Cultures were incubated at 37 °C under 5% CO_2_ in a humidified environment. To induce osteogenic differentiation, cells were seeded into culture dishes with a diameter of 18 mm at a density of 1.2 × 10^5^ cells/mL, with 6 × 10^4^ cells per dish. After 12 h of attachment, the medium was replaced with osteogenic medium containing 50 μg/mL ascorbic acid and 10 mM β-glycerophosphate. The cells were then assigned to the control, SMF, PEMF, and CEMF groups. The control group was maintained under the geomagnetic field environment, whereas the SMF, PEMF, and CEMF groups were exposed to the corresponding electromagnetic field stimulation for 20 min per day. The cells were subsequently cultured under the same conditions for differentiation experiments.

### 2.9. Alkaline Phosphatase (ALP) Staining

MC3T3-E1 cells were cultured in osteogenic induction medium for 7 days, followed by ALP staining. The cells were washed with PBS, fixed with 4% paraformaldehyde for 15 min, and rinsed three times. They were then incubated with BCIP/NBT solution at room temperature for 30 min. After three additional washes with PBS, images were acquired under a microscope, and the stained area was quantified.

### 2.10. Alizarin Red Staining

After 14 days of osteogenic induction, matrix mineralization in MC3T3-E1 cells was evaluated by Alizarin Red S staining (Servicebio, Wuhan, China). The cells were first washed with PBS, fixed in 4% formaldehyde for 15 min, and rinsed three times. Alizarin Red S solution was then added, followed by incubation at room temperature for 30 min. After three further washes with PBS, images were acquired under a microscope, and the average integrated optical density was quantified.

### 2.11. Transcriptome Sequencing and Data Analysis

MC3T3-E1 cells were cultured with CEMF system stimulation for 14 days, and the control group cells were not subjected to any stimulation. Total RNA was isolated using TRIzol reagent (Ambion, Carlsbad, CA, USA) and stored at −80 °C before sequencing. Transcriptomic data were processed in R (version 4.2.3). Differentially expressed genes were identified using Benjamini–Hochberg-adjusted P values, with significance defined as *p* < 0.05 and |log2 FC| > 0.5. Gene Ontology (GO) and Kyoto Encyclopedia of Genes and Genomes (KEGG) analyses were then performed for functional annotation and pathway enrichment.

### 2.12. Western Blot Analysis

Cells were lysed, and the supernatant was collected after centrifugation. Protein concentration was determined, and the samples were separated by SDS-PAGE followed by transfer onto PVDF membranes. After blocking with 5% nonfat milk for 3 h at room temperature, the membranes were incubated with primary antibodies against Runx2, COL-1, and OPN, followed by HRP-conjugated secondary antibodies. Protein bands were visualized using ECL and captured with a chemiluminescence imaging system (T5200Multi, Shanghai Peiqing Technology Co., Ltd., Shanghai, China).

### 2.13. Quantitative Real-Time PCR Analysis

Total RNA was extracted from treated cells. RNA isolation was performed using the TRIzol method, including cell lysis, phase separation, isopropanol precipitation, and ethanol washing. The purified RNA was dissolved in DEPC-treated water, and its concentration and purity were determined. cDNA was then synthesized according to the manufacturer’s instructions, followed by quantitative real-time PCR (qPCR) using a SYBR Green system. Target gene expression was normalized to GAPDH, and the primer sequences are listed in Table 1.

### 2.14. Statistical Analysis

All data are presented as mean ± standard deviation (SD). Statistical analysis was performed using GraphPad Prism 9 software (Version 9; GraphPad Software, San Diego, CA, USA). Intergroup differences were analyzed by one-way analysis of variance (ANOVA) followed by Tukey’s post hoc test or by nonparametric testing, as appropriate. A value of *p* < 0.05 was considered statistically significant.

## 3. Results

### 3.1. Theoretical Design and Numerical Simulation of CEMF

**Figure 1:**
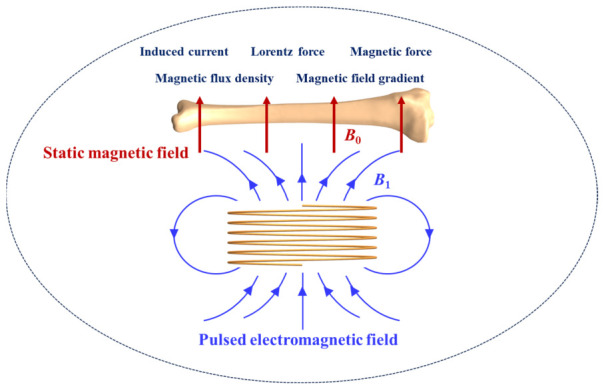
Principle diagram of the CEMF.

We first established a theoretical framework for the CEMF and performed numerical simulations to characterize its spatial magnetic field distribution and induced electromechanical effects. The cytoskeleton and mechanosensitive channels are core targets for mechanotransduction, and their effective activation requires mechanical stimulation in the order of 10 pN [21,22]. As shown in Figure 1, an SMF ($B_{0}$) is applied to bone tissue simultaneously with a PEMF ($B_{1}$) to form the CEMF. The time-varying magnetic field induces an electric field, which subsequently generates induced currents in bone tissue. Under the influence of the SMF, these currents give rise to Lorentz forces. The following is a theoretical analysis of the processes involved in generating magnetic, electric, and mechanical effects in the CEMF:

It is well established that materials experience a magnetic force when an SMF gradient exists [23]. Magnetic force is a major physical quantity associated with SMF exposure in biological tissues. [24]. It can be expressed as follows:

(1) $$F_{0}=\frac{\chi }{\mu_{0}}B_{0}\frac{\partial B_{0}}{\partial z}$$

where $F_{0}$ is the magnetic force in the direction of z, χ is the magnetic susceptibility of the subjects, $B_{0}\;$ is the magnetic flux density, and $\mu_{0}$ is the permeability of free space (which is a constant).

We propose exposing the subject to an alternating electromagnetic field generated by a solenoid coil powered by a sinusoidal dynamic current, as described in Equation (2), with the operating frequency range of 10 kHz to 5 MHz. This current generates dynamic electromagnetic fields within the object, as described in Equations (3) and (4), and simultaneously induces eddy currents J oscillating at the same frequency, as shown in Equation (5):

(2) I=N·A·sin(ωt)

(3) $$B_{1}=\oint \frac{\mu }{4\pi }\cdot \frac{Idl\times e_{r}}{r^{2}}$$

(4) $$\nabla \times E=-\frac{\partial B_{1}}{\partial t}$$

(5) J=σ·E

Here, I is the input current driving the coil, N stands for turns of the coil, A is the input current intensity, ω is the angular frequency, t represents the time; $B_{1}$ is the magnetic flux density of the alternating magnetic field, μ is the permeability, r is the distance between the tissue within the region of interest (ROI) and the elementary current Idl flowing on the coil, $e_{r}$ is the unit vector pointing toward the tissue; J is induced current density over the tissue, σ is the electrical conductivity of the tissue. Through observing these equations, we can find that the intensity of the induced current is in direct proportion to the input current intensity, frequency, turns per coil, and tissue conductivity.

When an additional SMF is introduced, the (***B***) can be written as follows:

(6) $$B=B_{0}+B_{1}$$

where $B_{0}$ represents the flux density of the SMF directed along the -z direction, whereas $B_{1}$ represents the flux density of the alternating magnetic field. Under these conditions, the Lorentz force (F) acts on the component of current density J that is perpendicular to both $B_{0}$ and ***B*_1_**, thereby generating mechanical stimulation in bone tissues.

The expression for Lorentz forces is expressed as:

(7) F= J×B

where F is the density of Lorentz exerted over the tissue, J represents the induced current density. According to Equation (7), the magnitude of the induced Lorentz force depends on the magnitudes of B and J, as well as the angle between them. The Lorentz force achieves its maximum when J and B are perpendicular. Considering the limited tolerance of tissues to electrical stimulation, enhancing the strength of the magnetic flux density $B_{0}$ and maintaining a perpendicular orientation emerges as an effective strategy to augment the Lorentz force [25].

**Figure 2:**
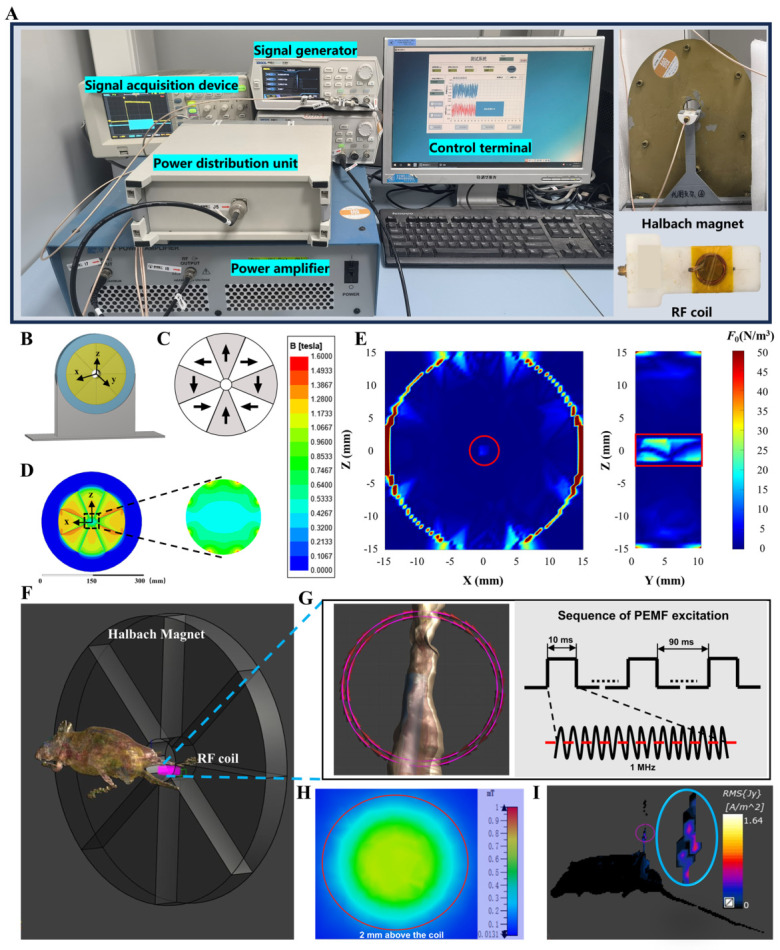
CEMF System and Spatial Distribution of Electromagnetic Multiphysics Parameters. (**A**) CEMF Devices. (**B**) Schematic of the Halbach magnet structure. (**C**) The arrows indicate the magnetization directions of the individual magnet blocks. (**D**) Radial cross-sectional distribution of magnetic flux density of Halbach magnet. (**E**) Static magnetic force density mapped in the hind limb of mice. The ROI in the figure is highlighted in red, indicating the region of the mouse skeleton subjected to CEMF. (**F**) Numerical simulation of a mouse model exposed to a Halbach magnet and PEMF coil. (**G**) Diagram of RF coil and input signal parameters. (**H**) Maximum Intensity Distribution of PEMF 2 mm above the coil. The ROI has been selected as a 30 mm diameter circular area and highlighted in red. (**I**) Distribution of induced current density in mouse legs.

Taking the mouse hind limb as a representative example, the comprehensive diagram in Figure 2A illustrates the experimental setup. To ensure simultaneous exposure to a perpendicular SMF and the induced current generated by the PEMF, the mouse hind limb was strategically placed within the central aperture of a Halbach magnet. The RF coil was positioned beneath the sample, parallel to the ground. When bone tissue was exposed to SMF or CEMF, the magnetic field direction was vertically upward (Figure 2C), with an onsite magnetic flux density of 0.4–0.6 T and a magnetic field gradient of 2.03 T/m (Figure 2D). The average magnetic force density exerted on the mouse hind limb was calculated to be 12.32 N/m^3^ (Figure 2E). The RF coil used for the PEMF has an inner diameter of 15 mm and 20 turns. The 1 MHz dynamic input current of the RF coil was modulated into square waves with a 10% duty cycle (Figure 2G). When the input current was set to 1 A, the maximum intensity of the PEMF generated by the RF coil in the target area (2 mm above the coil) was calculated as 0.38 ± 0.19 mT (Figure 2H). The average current densities in cortical bone, trabecular bone, and bone marrow of the mouse hind limb were 0.092 A/m^2^, 0.38 A/m^2^, and 0.48 A/m^2^, respectively (Figure 2I). The corresponding average resultant forces were 1.88 × 10^−9^ N, 3.86 × 10^−9^ N, and 4.77 × 10^−9^ N, respectively. Collectively, these simulations confirm the spatial distribution and magnitude of electromagnetic multiphysics parameters required to generate effective mechanical stimulation in vivo.

### 3.2. CEMF Exposure Mitigated Unloading-Associated Bone Loss and Improved Mechanical Performance in HLU Mice

**Figure 3:**
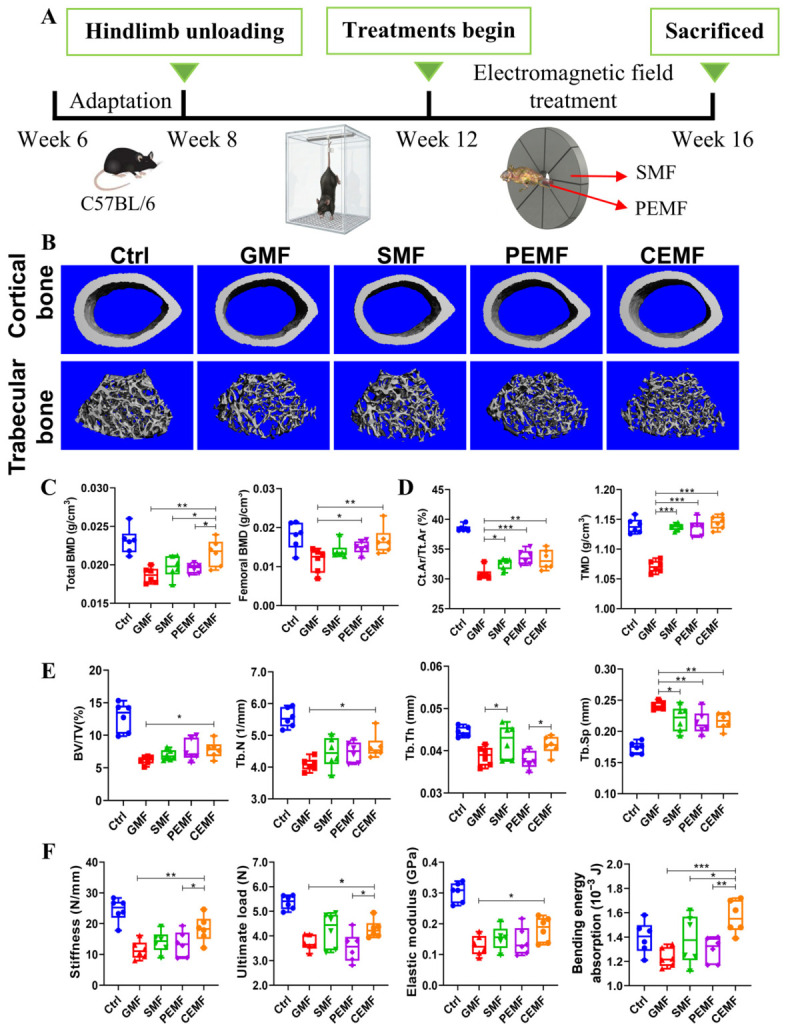
CEMF exposure enhanced bone mass and biomechanical performance in HLU mice. (**A**) Electromagnetic field intervention protocol in mice. (**B**) Micro-CT reconstruction of femoral cortical and trabecular bone, scale bar = 50 μm. (**C**) BMD of whole body and femur in mice was measured by DEXA. (**D**) Cortical indices, including Ct.Ar/Tt.Ar and TMD, were detected by three-dimensional images. (**E**) The trabecular parameters, including BV/TV, Tb.N, Tb.Th and Tb.Sp were detected by three-dimensional images. (**F**) Bone mechanical properties were detected by the three-point bending test, including stiffness, ultimate load, elastic modulus, and bending energy absorption. *n* = 6. Means ± SD; * *p* < 0.05, ** *p* < 0.01, *** *p* < 0.001.

Mechanical unloading was applied using hindlimb unloading (Figure 3A). Compared with control mice, HLU led to significant deterioration in both cortical and trabecular bone (Figure 3B), confirming the successful establishment of an unloading-related bone loss model. In the HLU model, we compared bone mass, bone microarchitecture, and mechanical properties following different electromagnetic stimulation treatments. DEXA analysis indicated that the CEMF group exhibited greater Total BMD than the GMF, SMF, and PEMF groups. For femoral BMD, both CEMF and PEMF groups were elevated relative to GMF, while SMF showed no notable difference (Figure 3C). Micro-CT analysis revealed that Ct.Ar/Tt.Ar and TMD were enhanced in SMF, PEMF, and the CEMF group compared with the GMF group (Figure 3D). Only the CEMF group showed increases in BV/TV and Tb.N among the evaluated trabecular parameters; Tb.Th was higher in CEMF than in PEMF and comparable to SMF, whereas Tb.Sp was reduced across SMF, PEMF, and CEMF groups (Figure 3E). Three-point bending tests showed that CEMF improved tibial mechanical properties, with Stiffness and Ultimate Load increased relative to PEMF, elastic modulus elevated in CEMF only, and bending energy absorption highest in CEMF compared with both SMF and PEMF groups (Figure 3F).

### 3.3. CEMF Enhanced Osteoblast Activity and Bone Formation Indices in HLU Mice

**Figure 4:**
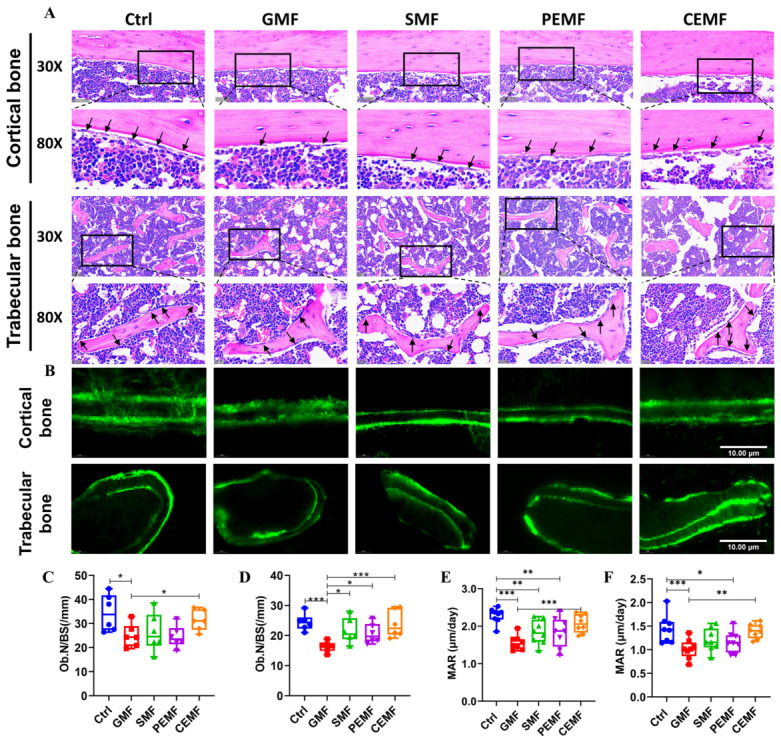
CEMF enhances bone formation in HLU mice. (**A**) H&E staining of femoral cortical bone (upper) and trabecular bone (lower). Scale bars: 50 μm for 30× images and 20 μm for 80× enlarged images. Arrows indicate osteoblasts. (**B**) Dynamic bone histomorphometric analysis based on double calcein labeling. Mice received two intraperitoneal injections of calcein 10 and 3 days before sample collection. The distance between the two fluorescent labels was used to calculate the MAR, reflecting new mineral deposition and dynamic bone formation activity during the 7-day labeling interval. Scale bar = 10 μm. (**C**) N.Ob/BS in cortical bone. (**D**) N.Ob/BS in trabecular bone. (**E**,**F**) New bone formation in cortical and trabecular bone. MAR was quantified using ImageJ (version 1.54g). *n* = 6. Means ± SD; * *p* < 0.05, ** *p* < 0.01, *** *p* < 0.001.

Bone loss caused by unloading is mainly associated with inhibition of bone formation mediated by osteoblasts. Therefore, we investigated the effects of CEMF on osteoblast distribution and bone formation in HLU mice. Osteoblast localization on cortical and trabecular bone surfaces was visualized by H&E staining (Figure 4A). Data analysis showed that CEMF significantly increased N.Ob/BS in cortical bone (Figure 4C). In contrast, SMF, PEMF, and CEMF treatments all increased N.Ob/BS in trabecular bone (Figure 4D). Furthermore, dynamic histomorphometric analysis revealed that CEMF significantly enhanced MAR in cortical (Figure 4E) and trabecular bone in HLU mice (Figure 4F), while SMF and PEMF groups showed no significant changes. As MAR is calculated from the distance between two fluorescent labels over the labeling interval, it reflects the rate of new mineral deposition. The increased MAR after CEMF treatment indicates that CEMF promoted bone formation.

### 3.4. CEMF Promoted Osteogenesis in MC3T3-E1 Cells

**Figure 5:**
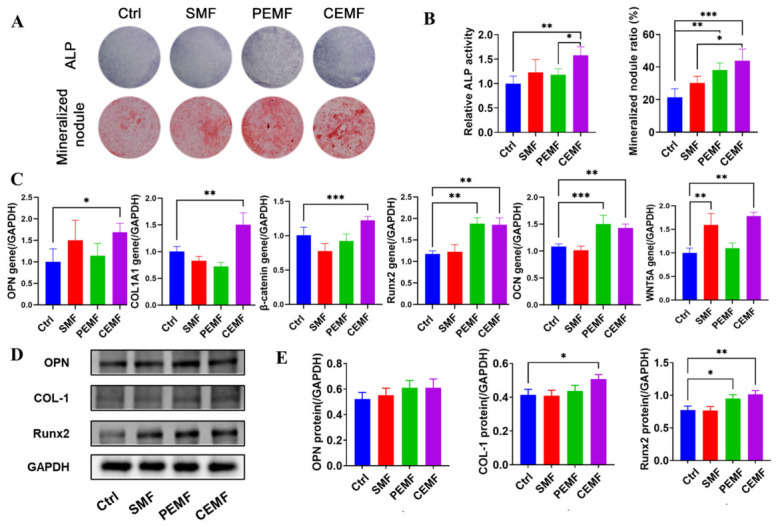
CEMF promotes osteogenesis in MC3T3-E1 cells. (**A**,**B**) ALP activity on day 7 and mineralized nodule formation on day 14. (**C**) mRNA expression of bone formation-related genes. (**D**,**E**) Protein expression of bone formation-related markers. *n* = 3. Means ± SD; * *p* < 0.05, ** *p* < 0.01, *** *p* < 0.001.

To examine the role of CEMF in osteogenic responses, we evaluated MC3T3-E1 cells under standard culture conditions. CEMF treatment enhanced ALP activity on day 7 of osteogenic induction, showing a stronger effect than PEMF. By day 14, both CEMF and PEMF increased mineralized nodule formation, with the CEMF group displaying the greatest area and degree of mineralization (Figure 5A,B). qRT-PCR analysis revealed that OPN, COL1A1, and β-catenin expression were elevated only in the CEMF group, whereas Runx2 and OCN were upregulated in both PEMF and CEMF groups. Moreover, CEMF promoted WNT5A expression in the osteogenic Wnt pathway. (Figure 5C). Western blot analysis showed no change in OPN protein among groups; however, COL-1 was elevated in the CEMF group relative to control, while Runx2 was increased in both PEMF and CEMF groups (Figure 5D,E).

### 3.5. CEMF Exposure Activated PI3K-AKT and ECM-Integrin Signaling Pathways in MC3T3-E1 Cells

**Figure 6:**
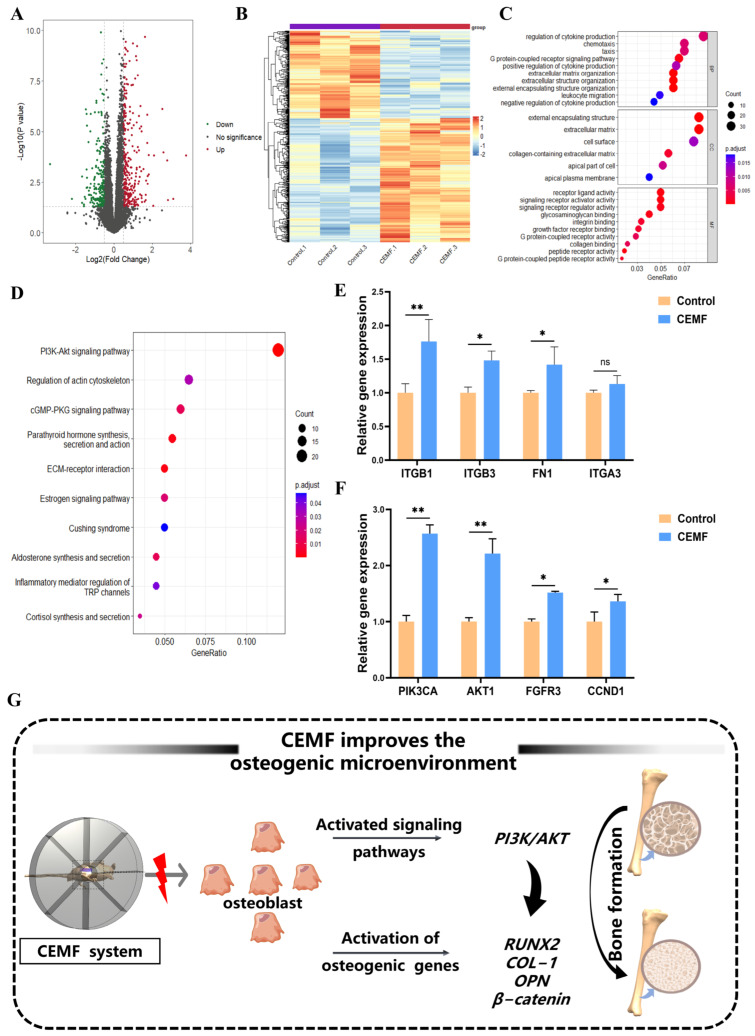
Effects of CEMF on the ECM-integrin-PI3K-AKT Signaling Pathway. (**A**,**B**) DEGs identified after CEMF treatment. (**C**) GO enrichment analysis including BP (biological process), CC (cellular component), and MF (molecular function). (**D**) KEGG enrichment analysis of osteogenesis-related pathways. (**E**) Effects of CEMF stimulation on mechanotransduction-related (IGTB1, ITGB3, FN1 and ITGA3) and (**F**) PI3K-AKT Signaling Pathway-related (PIK3CA, AKT1, FGFR3 and CCND1) gene expression. (**G**) Schematic diagram of the mechanism of CEMF-promoted bone formation. *n* = 3. Means ± SD; * *p* < 0.05, ** *p* < 0.01.

Based on the preceding in vivo and in vitro phenotypic results, CEMF exhibited a more favorable overall protective effect against unloading-induced bone loss and promoted osteogenic responses. Therefore, to further investigate the potential molecular mechanisms underlying CEMF-induced osteogenesis, RNA sequencing (RNA-seq) analysis was performed on osteoblasts from the control and CEMF-treated groups after 14 days of culture. RNA-seq analysis revealed 510 differentially expressed genes (DEGs) between the control and CEMF groups, including 299 upregulated and 211 downregulated genes (Figure 6A,B). GO analysis indicated that CEMF mainly affected biological processes associated with cytokine production, extracellular matrix organization, extracellular structure organization, external encapsulating structure organization, and G-protein-coupled receptor activity. Changes were also observed in cellular component and molecular function categories, particularly those related to the extracellular matrix, collagen-containing structures, and signaling receptor regulator activity (Figure 6C). KEGG enrichment analysis further showed that these DEGs were primarily associated with osteogenesis-related signaling pathways, with PI3K-AKT signaling being one of the most prominently enriched pathways. Enrichment was also evident in the ECM-receptor interaction and regulation of actin cytoskeleton pathways (Figure 6D). Notably, the expression of key mechanotransduction receptor genes including ITGB1, ITGB3, and the extracellular matrix bridging protein FN1 was significantly upregulated (Figure 6E), confirming that cells sense mechanical stimulation through the ECM-integrin. Subsequently, the coordinated upregulation of the PI3K catalytic subunit gene PIK3CA, the effector kinase gene AKT1, the upstream regulatory factor FGFR3, and the downstream cell cycle-related gene CCND1 indicated activation of the PI3K-Akt signaling pathway (Figure 6F).

## 4. Discussion

In mechanically unloading-related bone loss (e.g., microgravity-induced skeletal deterioration), there is a need for non-invasive physical countermeasures that can provide substitute mechanical input. Here, we evaluate a CEMF with particular attention to the resulting Lorentz-force-mediated mechanical stimulation. Although SMF and PEMF have been reported to benefit bone when applied separately, direct evidence for a role of the Lorentz force in regulating bone formation remains limited. Using an electromagnetic-mechanical-bone multi-field coupling framework and integrating theoretical analysis, numerical simulation, and in vivo and in vitro experiments, we show that, under the present parameters, CEMF provides more effective mechanical input and pro-osteogenic responses than single-field stimulation, supporting its potential as a complementary countermeasure for unloading-related bone loss.

Previous studies suggest that cellular mechanosensing—mediated by the cytoskeleton and mechanosensitive ion channels—can be triggered by forces on the order of ~10 pN [21,22]. In this study, theoretical analysis and numerical calculations indicated that the CEMF configuration generates Lorentz forces on the order of 10^−9^ N (~1000 pN) via field coupling, exceeding this commonly cited mechanosensing threshold by several orders of magnitude and supporting the capacity of CEMF to elicit mechanobiological responses at the cellular level. To better characterize the advantages of CEMF in this context, we compared it with other commonly used physical stimulation modalities. Ultrasound and vibration require direct contact with the skin, and the applied mechanical signals are readily attenuated, absorbed, or blocked by the overlying skin, muscle, and bone matrix [26]. By contrast, CEMF provides non-invasive mechanical stimulation that is not limited by tissue barriers. In the present study, the PEMF component was delivered with a carrier frequency of 1 MHz and a burst frequency of 10 Hz, and the magnetic flux density generated by the RF coil was below 0.4 mT. PEMF responses are known to depend strongly on frequency, waveform, and current dynamics, leading to marked variations in osteogenic outcomes [27,28,29,30]. Conventional PEMF mainly relies on electrical stimulation and is commonly applied for osteoporosis and fracture healing [31,32,33], typically employing low-frequency parameters established in the Bassett era (76.6 Hz, 1–15 Hz, or 3–5 kHz) [34,35]; however, electrical stimulation alone does not provide sufficient mechanical cues. To enhance the static field component, we used a Halbach array to concentrate magnetic field lines and generated a medium-intensity SMF of 0.4 T–0.6 T. Compared with the PEMF, this SMF showed a magnetic flux density about three orders of magnitude greater, together with a field gradient of 2.03 T/m. With an estimated osteoblast volume of 10^−15^ m^3^ [36], the magnetic force produced by this SMF was approximately 10^−2^ pN, which remains well below the mechanosensing threshold and is unlikely to trigger mechanotransduction. Achieving pN-level magnetic forces using SMF alone would require gradients ≥1 kT/m [37], which in turn would require superconducting magnets and involve substantial technical and economic constraints. These considerations indicate that SMF alone cannot provide adequate mechanical stimulation to osteoblasts, whereas the CEMF approach used here, by superimposing alternating and static magnetic fields, is able to generate Lorentz forces several orders of magnitude above the mechanosensing threshold within practical engineering limits, providing a clear physical basis for its ability to elicit cellular mechanobiological responses.

BMD and bone microarchitecture are critical indicators for evaluating osteoporosis. BMD reflects the mineral content of bone, whereas microarchitectural parameters provide insights into bone morphology. In this study, CEMF markedly promoted both bone mass and microstructural integrity in mice. DEXA analysis demonstrated that the CEMF group exhibited higher Total and Femoral BMD compared with the single-field groups (GMF, SMF, PEMF), highlighting its advantage in enhancing bone density. Consistently, micro-CT analysis confirmed that CEMF increased BV/TV and Tb.N, indicating superior improvements in trabecular microstructure. CEMF significantly improved tibial mechanical properties, indicating a reduced risk of fracture. Moreover, CEMF treatment increased osteoblast numbers and mineral apposition rates in cortical as well as trabecular bone, suggesting a further contribution to bone formation.

Osteoblasts are the principal mediators of bone formation, and CEMF exhibited a strong pro-osteogenic effect. qRT-PCR analysis showed that the osteogenic marker genes OPN, COL1A1, β-catenin, Runx2, and OCN were upregulated, confirming the role of CEMF in promoting osteogenic differentiation. The increased expression of WNT5A pointed to the involvement of Wnt signaling in CEMF-driven osteogenesis, coordinating osteoblast fate and function. Consistently, the CEMF group exhibited greater mineralized nodule area, higher mineralization degree, and enhanced ALP activity. Western blot analysis further supported the osteogenic effect of CEMF.

RNA-seq analysis showed that CEMF significantly affected PI3K-Akt signaling and ECM-receptor interaction-related pathways. GO analysis also demonstrated significant enrichment in ECM, suggesting that these pathways play critical roles in CEMF-induced osteogenesis. PI3K-AKT is a key pathway regulating cell survival, proliferation, and protein synthesis, and its role in osteogenesis has been confirmed by multiple studies [38,39]. qRT-PCR results confirmed the upregulation of key PI3K-AKT-related components (FGFR3, PIK3CA, AKT1, CCND1). The ECM-integrin axis represents a central mechanotransduction pathway, mediating cellular perception and transmission of external mechanical cues through interactions between the extracellular matrix and membrane receptors. This is consistent with previous studies identifying integrin complexes as crucial mechanosensitive units in force transmission [40,41]. Recent evidence shows that TGF-β1 can promote mesenchymal stem cell osteogenesis by inducing an integrin-mediated mechanical positive autoregulation loop along the integrin-FAK-YAP axis, highlighting the broader relevance of integrin-dependent mechanotransduction in bone formation [42]. Substantial evidence has also demonstrated that the PI3K-Akt pathway is mechanosensitive and responsive to multiple forms of mechanical stimulation, including fluid shear stress [43,44]. qRT-PCR results demonstrated significant upregulation in the gene expression of FN1, a key ECM component, and the core mechanosensitive receptors ITGB1 and ITGB3. The upregulation of FN1 enhances the mechanical signaling capacity of the ECM, while integrins (ITGB1/ITGB3), as transmembrane bridges and mechanoreceptors, mediate the transmission of external mechanical signals anchored in the ECM into the intracellular environment [45]. Therefore, the mechanical stimuli generated by CEMF are likely transmitted through the ECM-integrin-mediated mechanotransduction mechanism, which activates downstream signaling pathways such as PI3K-Akt, thereby promoting osteogenesis.

## 5. Conclusions

In conclusion, this study shows that the CEMF provides coupled multiphysics input, including mechanically relevant stimulation, and enhances osteogenic responses via ECM–integrin-mediated mechanotransduction with engagement of PI3K–AKT signaling, thereby mitigating unloading-related bone loss. CEMF improved bone mass, bone microarchitecture, and mechanical properties and increased bone formation indices in HLU mice; it also promoted osteogenic differentiation and upregulated osteogenesis-related genes in MC3T3-E1 cells, with transcriptomic data further supporting the involvement of these mechanotransduction pathways. These findings indicate that CEMF may serve as a non-invasive physical countermeasure for spaceflight-associated bone loss.

## 6. Patents

(1) Frequency-adaptive control method, system, magnetic therapy device, and readable storage medium, China, Patent No. ZL202210326911.8, granted; valid from 2022-03 to 30 to 2042-03-30 [46].

(2) Dynamic magnetic field generation method, apparatus, device, and computer-readable storage medium, China, Patent No. ZL202210323630.7, granted; valid from 2022-03 to 30 to 2042-03-30 [47].

## Figures and Tables

**Table 1:** Primer sequences used for qRT-PCR analysis.

Genes	Primer Sequence (5′ to 3′)	
OPN	F: TCTCCTTGCGCCACAGAATG	R: GTGGTCATGGCTTTCATTGGA
COL1A1	F: CACTGCAAGAACAGCGTAGC	R: AAGTTCCGGTGTGACTCGTG
β-catenin	F: CAGTGGGATGGTGGGTGTAAG	R: CAGGAAGGGATGGAAGGTCTC
Runx2	F: TAGCCAGGTTCAACGATCTGA	R: GCTTCTGTCTGTGCCTTCTTGG
OCN	F: TTCTGCTCACTCTGCTGACCCT	R: CCTGCTTGGACATGAAGGCTT
WNT5A	F: TCATGAACTTGCACAACAATGA	R: CCGTCTTAAACTGGTCATAGCC
ITGB1	F: CGGCCAGAAGACATTACTCAG	R: GGGTAATCTTCAGCCCTCTTG
ITGB3	F: CTCTGTGGCCAGTGTGTCTG	R: TAGCCAGTCCAGTCCGAGTC
FN1	F: CTTGCACGATGATATGGAGA	R: AGCTGAACACTGGGTGCTAT
ITGA3	F: CTGGTGCCTACAACTGGAAA	R: AGGTTTCCTTGCTCCTCTGA
PIK3CA	F: AAAATGGCGACGACTTACGG	R: GGCCTTGGTTTTGCCAGATG
AKT1	F: CCGCCTGATCAAGTTCTCCT	R: AGATGATCCATGCGGGGCTT
FGFR3	F: GATCATGCGGGAATGTTGGC	R: AGGTCCAAGTACTCGTCGGT
CCND1	F: TGGAGCCCCTGAAGAAGAG	R: AAGTGCGTTGTGCGGTAGC
GAPDH	F: TGCACCACCAACTGCTTAG	R: GGATGCAGGGATGATGTTC

## Data Availability

The original contributions presented in this study are included in the article. Further inquiries can be directed to the corresponding authors.
